# Gender differences in the antianhedonic effects of repeated ketamine infusions in patients with depression

**DOI:** 10.3389/fpsyt.2022.981981

**Published:** 2022-09-16

**Authors:** Wei Zheng, Xin-Hu Yang, Li-Mei Gu, Jian-Qiang Tan, Yan-Ling Zhou, Cheng-Yu Wang, Yu-Ping Ning

**Affiliations:** ^1^The Affiliated Brain Hospital of Guangzhou Medical University, Guangzhou, China; ^2^The First School of Clinical Medicine, Southern Medical University, Guangzhou, China

**Keywords:** ketamine, depression, gender differences, anhedonia, response

## Abstract

**Objectives:**

Subanaesthetic ketamine (0. 5 mg/kg/40 min intravenous infusion) produces rapid and robust antianhedonic effects in subjects with mood disorders, independent of other depressive symptoms. The objective of this study was to examine potential differences in rate of antianhedonic response to ketamine in males and females, which has not been previously examined.

**Methods:**

A total of 135 patients with depression (68 males, 67 females) who received six intravenous infusions of ketamine (0.5 mg/kg/40 min) during 2 weeks were enrolled. The anhedonia subscale of the Montgomery–Åsberg Depression Rating Scale (MADRS) was utilized to measure anhedonic symptoms. Antianhedonic remission and response were defined as ≥75 and ≥50% improvement of anhedonic symptoms at 24 h after the sixth ketamine infusion (day 13).

**Results:**

Antianhedonic response (50 vs. 47.8%, *p* > 0.05) and remission (26.5 vs. 14.9%, *p* > 0.05) rates did not differ significantly between males and females. A linear mixed model revealed a nonsignificant between-group difference in MADRS anhedonia subscale scores [F_(1, 132.5)_ = 1.1, *p* = 0.30]. Females reported a significantly larger reduction in anhedonic symptoms than males at the 2-week follow-up (*p* < 0.05).

**Conclusion:**

The rates of antianhedonic response and remission to multiple ketamine infusions for the treatment of depression were similar between males and females. These findings should be verified by future studies, preferably randomized controlled trials (RCTs).

## Introduction

Major depressive disorder (MDD) is a multisymptom condition that accounts for 40.5% of disability-adjusted life years (DALYs) caused by mental and substance use disorders (1), and females have from a twofold higher risk of MDD than males (2). Numerous studies have observed differences in clinical presentation and comorbidities between females and males with MDD (2, 3). For example, females were likely to have greater depressive symptom severity, earlier onset of first-episode MDD, and longer duration of depressive episodes than males in the Sequenced Treatment Alternatives to Relieve Depression (STAR^*^D) study (3). Alcohol and drug abuse and obsessive compulsive disorder were more common in males than in females (3).

Interestingly, females with MDD were significantly more likely than males to receive antidepressants (2). However, findings on gender differences in outcomes (i.e., response and remission rate, time to response and remission, and adverse drug reactions) of treatment with antidepressants were inconsistent. For example, an increasing number of studies have reported that females are more likely than males to have a positive response to antidepressants, especially selective serotonin reuptake inhibitors (SSRIs) (4–6). Other studies (4, 7) found a significantly greater therapeutic response to the tricyclic antidepressant (TCA) imipramine in males than in females.

The rapid antidepressant response to ketamine (0.5 mg/kg/40 min intravenous infusion), a glutamate N-methyl-D-aspartic acid (NMDA) receptor antagonist for individuals with treatment-resistant depression (TRD), suggests a possible new approach in the treatment of MDD and bipolar depression, which compares favorably to the multiple weeks required for current first-line pharmacotherapies (8–11). Furthermore, both single dose (12–15) and repeated dose (15–17) intravenous ketamine treatments exhibited rapid and sustained antisuicidal and antianhedonic effects. Importantly, Lally et al. found that ketamine's antianhedonic effects are independent of other depressive symptoms (12).

Sex differences in antidepressant response to intravenous ketamine treatment for patients with depression have been investigated, but with inconsistent findings (18). For example, Freeman et al. found no significant difference in antidepressant response to intravenous ketamine infusions in females suffering from TRD, as compared with males with this diagnosis (18). However, a recent systematic review and meta-analysis (437 participants receiving ketamine) found that males appeared to have slightly longer antidepressant responses to a single-dose administration of ketamine than females (19). However, there has been no testing for sex differences in antianhedonic response to ketamine infusions in patients with depression.

In this study we aimed to investigate the impact of sex on the antianhedonic effects of six infusions of 0.5 mg/kg ketamine over two weeks in Chinese patients with MDD or bipolar depression. Based on the findings of Freeman et al.'s study (18), we hypothesized that there is no difference in the efficacy of six ketamine infusions for ameliorating anhedonia levels between females and males with depression.

## Methods

This prospective cohort study of consecutive depressed patients with TRD and/or suicidal ideation treated at the Affiliated Brain Hospital of Guangzhou Medical University was initiated in November 2016. The current paper reports on results to date in an ongoing study. This study protocol (Clinical Trials Identifier: ChicCTR-OOC-17012239) was approved by the local ethics committee in accordance with the Declaration of Helsinki. All subjects signed written informed consent.

### Patients

The selection of patients has been previously described (20, 21). Briefly, all patients (68 males, 67 females) met the following inclusion criteria: (1) a major depressive episode at the beginning of this study that met the Diagnostic and Statistical Manual of Mental Disorders 5th edition (DSM-5) criteria for bipolar disorder or MDD diagnosis without hallucinations or delusions; (2) aged 18 to 65 years, Han Chinese; (3) a baseline Hamilton Depression Rating Scale-17 (HAMD-17) score ≥ 17; and (4) inadequate response to two or more sufficient courses of antidepressants and/or a baseline Scale for Suicidal Ideations (SSI)-part I score ≥ 2. Patients experiencing TRD and/or suicidal ideation who had a history of alcohol or substance dependence or major medical or neurological diseases (i.e., cancer or infectious disease) were excluded from this study.

### Intravenous injections of ketamine

In line with the methodology of previous studies (22, 23), all participants received six intravenous infusions of ketamine during 2 weeks. A detailed description of repeated-dose ketamine infusions in this study has been published in previous studies (20, 21). Briefly, following an overnight fast, all subjects received six intravenous infusions of ketamine hydrochloride administered over a 2-week period (3 times per week) by an intravenous pump. As recommended previously (23), the dose of ketamine was 0.5 mg/kg administered intravenously over a 40-min period. A trained psychiatrist recorded blood pressure, respiratory rate, and pulse frequency for all participants at 10-min intervals during and after each intravenous injection. All participants continued using their previously prescribed psychotropic medications throughout the study.

### Anhedonic symptoms

Clinical ratings of anhedonic symptoms were measured by clinical psychiatrists at baseline (day 0), at 4 and 24 h after each infusion of ketamine (0.5 mg/kg over 40 min) and at 2 weeks postinfusion (day 26) using the anhedonia item of the Montgomery–Åsberg Depression Rating Scale (MADRS). The anhedonia item of the MADRS included the following 5 items: inability to feel, concentration difficulties, lassitude, apparent sadness, and reported sadness, which has been utilized in previous studies and proven useful in evaluating anhedonia symptoms (24–26). A change in the MADRS anhedonia subscale scores from day 0 to 26 was the primary endpoint of this study. The secondary outcomes were as follows: antianhedonic remission rate (defined as a ≥75% reduction in MADRS anhedonia subscale scores at day 13) and response rate (defined as a ≥50% reduction in MADRS anhedonia subscale scores at day 13). For multiple assessments of the MADRS anhedonia subscale scores, the interrater correlation coefficient was > 0.9.

### Statistical analysis

SPSS version 24.0 (SPSS Inc., Chicago, United States) was used for all statistical analyses. For descriptive analyses, quantitative and qualitative variables were expressed as the means ± standard deviation (SD) and numbers (percentage) in both the male and female groups. We used Student's *t*-test and/or the Mann–Whitney *U*-test for continuous variables (which included education, age of onset, and duration of illness) and the χ^2^ test for categorical variables (which included gender, marital status, family history of psychiatric disorders) to compare the differences in demographic and clinical variables of the male and female groups. The rates of antianhedonic response and remission by gender were analyzed by χ^2^ test. Then, we compared the rates of antianhedonic response and remission by gender using odds ratios derived from logistic regression analyses after adjusting for the related variables. We compared the changes in MADRS anhedonia subscale scores from day 0 to 26 between the male and female groups using a linear mixed model after controlling for baseline level. Bonferroni corrections were utilized for multiple tests. A *P* < 0.05 was considered statistically significant.

## Results

As shown in Table 1, male patients with depression were more likely to be unmarried (*p* = 0.01, Bonferroni corrected *p* < 0.05/7 = 0.007) and living alone (*p* = 0.005, Bonferroni corrected *p* < 0.05/7 = 0.007) than female patients with depression. Male patients with depression had a higher body mass index (BMI) (*p* = 0.003, Bonferroni corrected *p* < 0.05/7 = 0.007), longer duration of illness (*p* = 0.009, Bonferroni corrected *p* < 0.05/7 = 0.007), younger age of onset (*p* = 0.005, Bonferroni corrected *p* < 0.05/7 = 0.007), more family history of psychiatric disorders (*p* = 0.009, Bonferroni corrected *p* < 0.05/7 = 0.007) and more history of psychiatric hospitalization (*p* = 0.01, Bonferroni corrected *p* < 0.05/7 = 0.007) than female patients with depression. After Bonferroni corrections, living alone, BMI, and younger age of onset remained significant (all *p* < 0.007).

**Table 1:** Comparison of demographic and clinical characteristics between male and female patients with depression.

**Variables**	**Male (*****n*** = **68)**		**Female (*****n*** = **67)**		**Statistics**		
	**N**	**%**	**N**	**%**	**χ^2^**	**df**	***P***
Married	32	47.1	46	68.7	6.5	1	**0.01**
Employed	26	38.2	26	38.8	0.01	1	0.95
Living alone	10	14.7	1	1.5	7.9	1	**0.005**
No history of psychiatric hospitalization	40	58.8	53	79.1	6.5	1	**0.01**
Having a family history of psychiatric disorders	32	47.1	20	29.9	4.2	1	**0.04**
Antianhedonic responders	34	50.0	32	47.8	0.1	1	0.80
Antianhedonic remitters	18	26.5	10	14.9	2.7	1	0.09
	Mean	SD	Mean	SD	T/Z	df	*P*
Age (years)	33.7	11.0	35.9	12.4	−0.9	—^a^	0.37
Education (years)	12.0	3.1	12.4	3.4	−0.7	133	0.46
BMI (kg/m^2^)	23.5	3.6	21.7	3.2	3.1	133	**0.003**
Age of onset (years)	23.3	10.4	29.1	11.9	−2.9	—^a^	**0.004**
Duration of illness (months)	122.4	99.9	81.3	78.3	2.7	133	**0.009**
Baseline HAMD-17 scores	23.0	4.6	24.6	5.5	−1.8	133	0.07
Baseline MADRS scores	32.2	7.4	33.4	8.4	−0.9	133	0.36
Baseline MADRS anhedonia subscale score	20.9	4.9	19.9	4.5	1.1	133	0.25

^a^Mann–Whitney U-test.

Bolded values are p < 0.05. BMI, body mass index; HAMD, Hamilton Depression Rating Scale; MADRS, Montgomery–Åsberg Depression Rating Scale.

Antianhedonic response rates were 50% (34/68) in male patients with depression and 47.8% (32/67) in female patients with depression. In terms of antianhedonic remission rates, male patients with depression reached 26.5% (18/68), and female patients with depression reached 14.9% (10/67). Antianhedonic response and remission rates did not differ significantly between the two groups (all *p* > 0.05). After controlling for confounders, there were still no significant differences (all *p* > 0.05).

**Figure 1:**
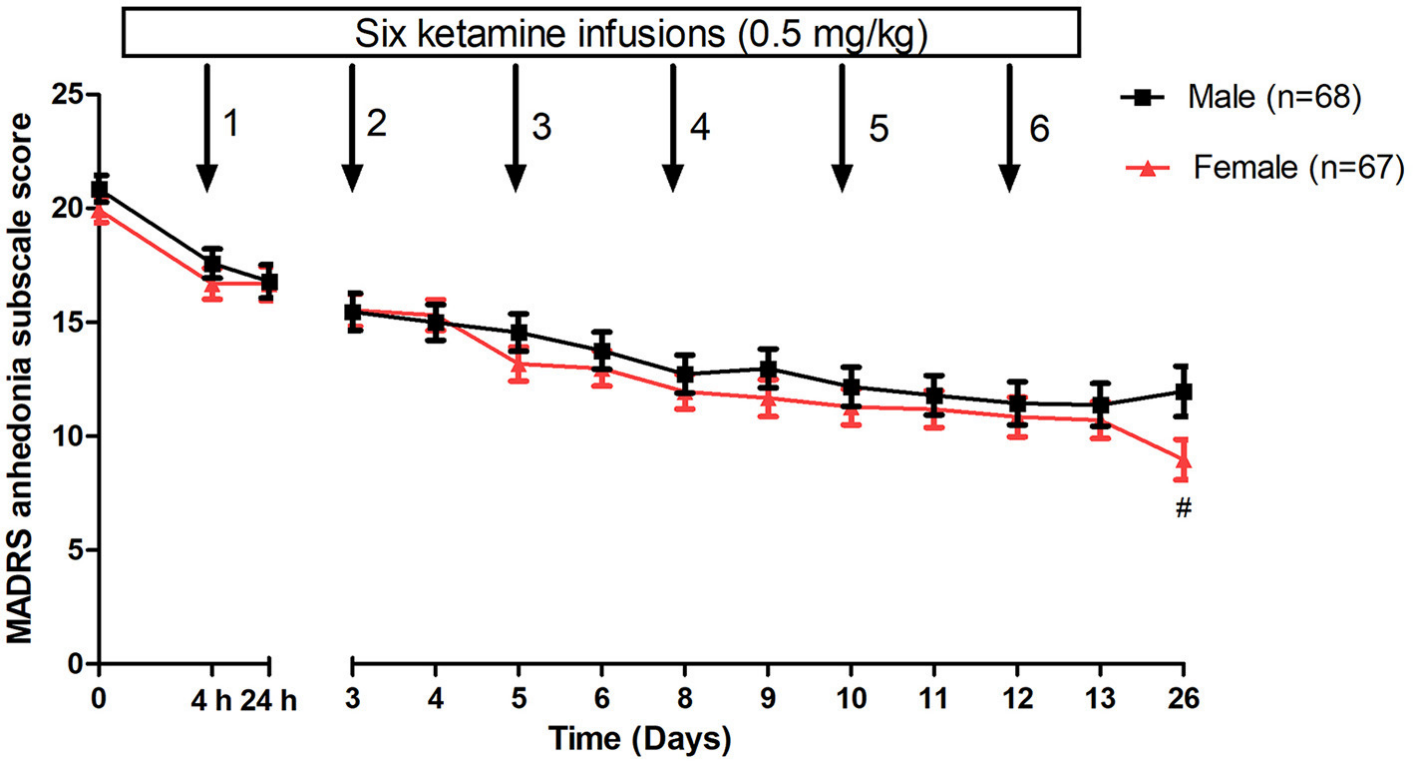
Gender differences in changes in anhedonia symptoms in patients with depression following multiple ketamine infusions. All values are presented as mean ± standard error. ^#^A significant difference was found at a given time point between women and men with depression (*p* < 0.05). MADRS, Montgomery-Åsberg Depression Rating Scale.

When analyzing the change in anhedonic symptoms over time, a nonsignificant between-group difference was found using a linear mixed model (time: F_(13, 1684.8)_ = 74.6, *p* < 0.001; group: F_(1, 132.5)_ = 1.1, *p* = 0.30; group-by-time interaction: F_(13, 1684.8)_ = 1.7, *p* = 0.05). When compared to baseline, as shown in Figure 1, a significant reduction in anhedonic symptoms was observed from the 1st to 6th injection as well as on day 26 in both groups (all *p* < 0.05). The subgroups did not significantly differ in the improvement of anhedonic symptoms from the 1st to 6th injection (Figure 1). Females were significantly associated with a greater reduction in anhedonic symptoms than males at day 26 (*p* < 0.05) (Figure 1).

## Discussion

To the best of our knowledge, this is the first study to determine the gender differences in the antianhedonic effects of ketamine (0.5 mg/kg/40 min intravenous infusion) in female vs. male patients with depression. The main findings of this study included the following: (1) six intravenous infusions of ketamine used in patients with depression are a similarly effective treatment in rapidly ameliorating anhedonia levels for both females and males; and (2) significantly lower MADRS anhedonia subscale scores were found only at the 2-week follow-up in females than in males after receiving six intravenous infusions of ketamine.

Although females appeared to have a significantly higher rate of anhedonia than males (27), no significant gender differences in antianhedonic response and remission rate were found in either females or males after receiving serial ketamine treatments in this study, which was similar to the findings of several studies examining the gender differences in the antidepressant effects of ketamine and esketamine (18, 28). For example, Jones et al. found that the antidepressant effects of esketamine nasal spray are similar in females vs. males suffering from TRD (28). Gender differences in outcomes for the use of other antidepressants such as SSRIs have been investigated, but with mixed findings (4, 5, 7, 29). For example, no gender differences in antidepressant efficacy were reported in some studies (30, 31). However, several studies reported that females responded better than males to SSRIs (5, 29), which was contrary to the findings of previous studies (4, 7). The inconsistent findings across the above studies may be due in part to differences in study design, study drug, and the inclusion criteria of participants.

Although no gender differences in the rates of antianhedonic response and remission to ketamine in patients with depression were found in this study, accumulating studies have found that various factors, including differences in hormone levels, drug metabolism and neuronal circuitry, may account for the disparity in treatment outcomes to other antidepressants between females and males. For example, many studies (32, 33), but not all (34–36), found that both sex hormone therapy and menopausal status were associated with treatment outcomes for antidepressants in females. A recent study found that the antidepressant effects of ketamine were not associated with menopausal status among women (18).

Several limitations are worth considering. First, the sample size of these analyses was relatively small, partly interpreting the negative findings on the rates of antianhedonic response and remission to ketamine in males versus females. Second, this was an open-label real-world clinical study rather than a randomized controlled trial (RCT). Furthermore, psychotropic medications might potentially affect the antianhedonic effects of ketamine. Third, although several animal studies reported that sex hormones might be related to ketamine treatment response (37, 38), hormone levels such as female oestradiol and female progesterone were not collected in this study. Finally, the current study was a *post hoc* secondary analysis focusing on patients with MDD and bipolar depression.

## Conclusion

The rates of antianhedonic response and remission to multiple ketamine infusions for the treatment of depression in males vs. females were similar. These findings should be verified by future RCTs with relatively large sample sizes.

## Data availability statement

The raw data supporting the conclusions of this article will be made available by the authors, without undue reservation.

## Ethics statement

The studies involving human participants were reviewed and approved by the Affiliated Brain Hospital of Guangzhou Medical University. Written informed consent to participate in this study was provided by the participants' legal guardian/next of kin.

## Author contributions

Y-PN: study design and critical revision of the manuscript. WZ, Y-LZ, and C-YW: data collection. WZ, X-HY, and L-MG: analysis and interpretation of data. WZ and J-QT: drafting of the manuscript. All the authors: approval of the final version for publication.

## Funding

This study was funded by the National Natural Science Foundation of China (82101609), Scientific Research Project of Guangzhou Bureau of Education (202032762), Science and Technology Program Project of Guangzhou (202102020658), the Science and Technology Planning Project of Liwan District of Guangzhou (202004034), Guangzhou Health Science and Technology Project (20211A011045), Guangzhou Science and Technology Project of Traditional Chinese Medicine and Integrated Traditional Chinese and Western Medicine (20212A011018), China International Medical Exchange Foundation (Z-2018-35-2002), Guangzhou Clinical Characteristic Technology Project (2019TS67), and Science and Technology Program Project of Guangzhou (202102020658). The funders had no role in study design, data collection and analysis, decision to publish, or preparation of the manuscript.

## Conflict of interest

The authors declare that the research was conducted in the absence of any commercial or financial relationships that could be construed as a potential conflict of interest.

## Publisher's note

All claims expressed in this article are solely those of the authors and do not necessarily represent those of their affiliated organizations, or those of the publisher, the editors and the reviewers. Any product that may be evaluated in this article, or claim that may be made by its manufacturer, is not guaranteed or endorsed by the publisher.
